# Keeping the Traction on in Orthopaedics

**DOI:** 10.7759/cureus.10034

**Published:** 2020-08-25

**Authors:** Baseem Choudhry, Billy Leung, Elizabeth Filips, Kawaljit Dhaliwal

**Affiliations:** 1 Trauma & Orthopedics, Maidstone and Tunbridge Wells NHS Trust, Tunbridge Wells, GBR; 2 Trauma & Orthopaedics, Royal Berkshire NHS Foundation Trust, Reading, GBR; 3 Trauma & Orthopaedics, King's College NHS Foundation Trust, London, GBR; 4 Orthopaedics, Maidstone and Tunbridge Wells NHS Trust, Tunbridge Wells, GBR

**Keywords:** traction, skeletal traction, bryant's traction, thomas splint, halo traction, hamilton-russell traction

## Abstract

The trauma and orthopaedic speciality continues to advance as surgery becomes more accessible and safe. However, the bygone days of treatment with traction still has its merits and should remain a part of practitioner's repertoire. This will allow the practitioners to be resourceful in times of unexpected scenarios.

We aim to write this article to describe indications, applications of various forms of traction, and their relevant complications.

## Introduction and background

Introduction

Traction is one of the oldest principal tenets of treatment in orthopaedics. The use of traction dates back as far as 3000 years in ancient Egypt [1]. Transcripts from ‘the father of medicine’, Hippocrates discuss about forces of extension and counter-extension, surmounting to traction [2]. However, as advances in surgical techniques, safety of surgery, high nursing requirements and the increasing economic pressures to reduce the length of hospital stay, the application of traction has been utilised less over time and consequently, the once-familiar skill of application of traction and its patient care has declined rapidly, becoming a dying art [3,4]. The aim of this article is to discuss the principles and provide instruction to the practitioner on how to apply common/various forms of traction appropriately.

Principles of Traction

Tractions' main goals are to control pain from muscle spasm, reduce fractures maintaining anatomical reduction, and to prevent and correct deformity. An effective traction will provide a pulling force on the body by ensuring a good grip on the injured limb that is adequate and secure. The traction and counter-traction forces must be in opposite directions. Splints and slings should be suspended without interference, ropes must slide freely through each pulley, line and magnitude of pull must be in line, precise weight must be applied and should be hanging free. A well-applied traction will achieve these objectives and hence reduce the risk of developing complications [3].

Types of Traction

1. Manual: applying the pull manually with the hands

2. Skin: applying the force over a large area of skin/soft tissue to transmit traction to the bone.

3. Skeletal: applying the force directly to the bone through metal pins inserted through the bone. Two additional methods for skin and skeletal traction:

(a) Fixed: the pull is between two fixed points.

(b) Sliding: the pull is exerted by a pull between hanging weights and the patient's own body weight [5].^ ^

Expected times for treatment

Total fracture healing time in weeks is the age of child plus two, early callus formation is one-third of total healing time, and rehabilitation time is two to four weeks [3].

Knots

**Figure 1:**
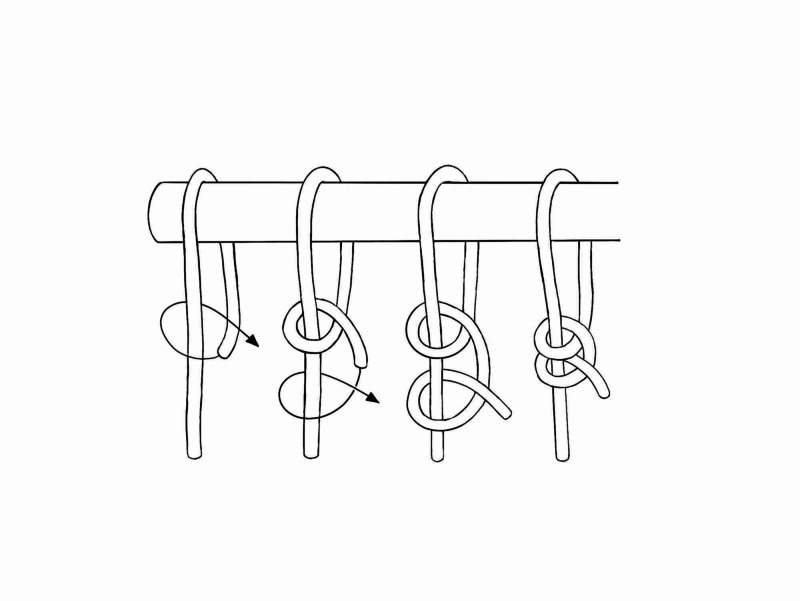
Two Half-Hitch Knot Illustration courtesy B. Leung.

Knots are used to secure traction cord to the end of the bed or frame. Ideal knots applied in traction are knots that can be tied with one hand whilst holding weight, easy to tie and untie, and will not slip. Royal College of Nursing (RCN) guidance recommends the two half hitches knot (Figure 1); however, other knots that can be considered include the clover hitch, barrel hitch, half hitch and reef knot. It is recommended that the cord is not reused due to wear and infection risk. The cords should be short and bound back on themselves with adhesive tape which prevents fraying of the cord end [3-5]. 

## Review

Femur

Skin Traction

Skin traction is the commonest and most popular form of traction used. It is utilised for the temporary management of fractures of the femoral neck and shaft in children, and post-reduction of native hip dislocation.

**Figure 2:**
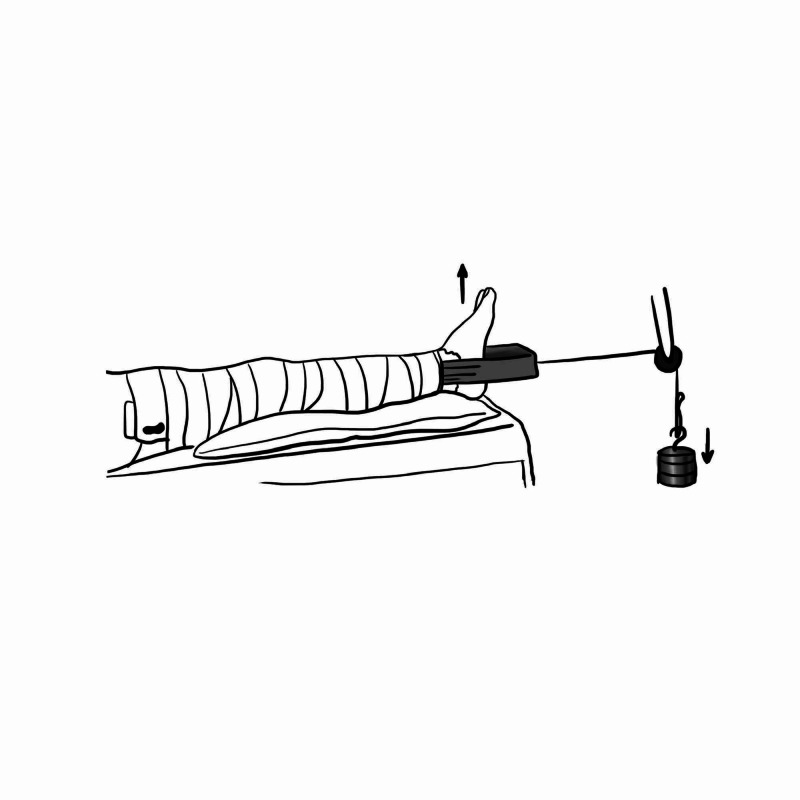
Skin traction Illustration courtesy E. Filips

Its application requires a non-adhesive tape that is applied on either side of the injured limb, ensuring the pressure areas are well padded; in this case, this is over the head of the fibula to prevent the development of common peroneal nerve neuropraxia, and avoid bandaging the malleoli and Achilles tendon (Figure 2). Approximately four fingers breath slack is left from the sole of the foot to allow for free dorsi- and plantar flexion and then bandaged firmly with a crepe bandage. The knee does not need to be bandaged to allow for visual assessment of leg alignment. This is then tied to the frame of bed with weights no more than 4.5 kg adjusted to patient weight [3]. 

Thomas Splint

**Figure 3:**
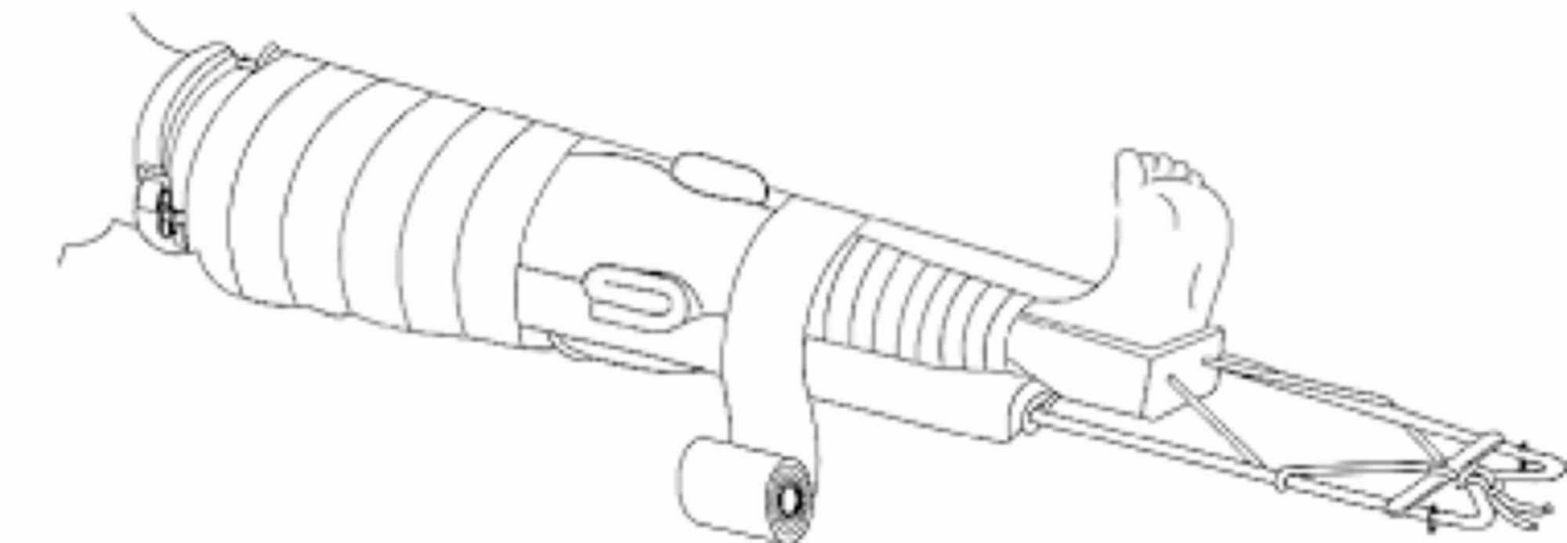
Thomas Splint Illustration reproduced with permission from Össur [7].

Named after early bone setter, Hugh Owen Thomas, (1834-1891) who pioneered the splint which reduced sequelae of a femur fracture saving lives during the First World War, the Thomas splint is a long leg splint with a hoop that extends beyond the foot which can be fixed or as part of balanced skin traction (Figure 3) [6]. 

The application of the splint involves measuring the uninjured limb length and the splint length is adjusted accordingly by adding another 15-20 cm; the circumference of the unaffected thigh is also measured and splint ring is sized to be more than 5 cm. Once adjusted, the slings are positioned along the splint to support the injured leg and the ring should fit into the groin and abut against the ischial tuberosity; this can be padded to protect from developing pressure sores.

Non-adhesive tape is applied to leg as described earlier which is then placed in the splint. Once the leg is placed in a splint, traction cords attached to the adhesive are then looped around the lateral and medial bar of the splint and then knotted to the end of the splint to prevent slipping, a windlass is applied to increase the traction force to the limb. This is now working as fixed traction [3]. 

*Hamilton-Russell Traction* 

**Figure 4:**
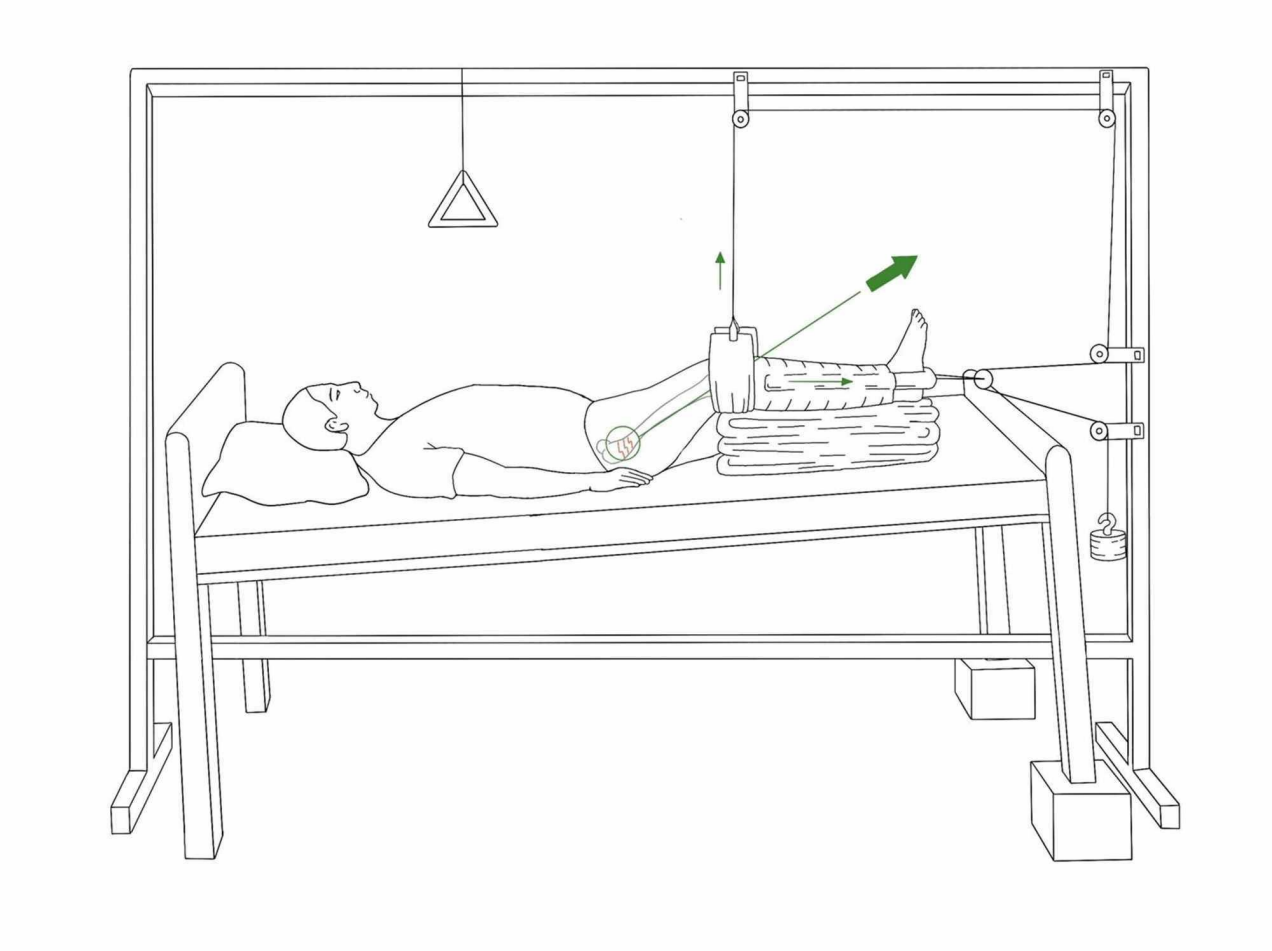
Hamilton-Russell skin traction Illustration courtesy B. Leung

A balanced traction system originally developed for the fracture of the femur to control muscle spasms, can also be used for acetabular fractures. Its set-up with Balkan beam frame with a crossbar above the knee, and two extension bars with crossbars at the foot end of the bed (Figure 4). 

A broad soft sling is placed under the knee that provides an upward force, which controls the posterior angulation of the distal fragment. Distal to the knee, skin traction (as described) is applied where the horizontal pull is on the tibia using cord, pulleys and weights. The mechanical forces are such that the horizontal pull is twice that of vertical pull which provides a resultant vector in line of axis of the femur. The traction cord is attached to sling and passes through the pulleys, which is balanced with a counterweight of approximately 3.5 kgs.

**Figure 5:**
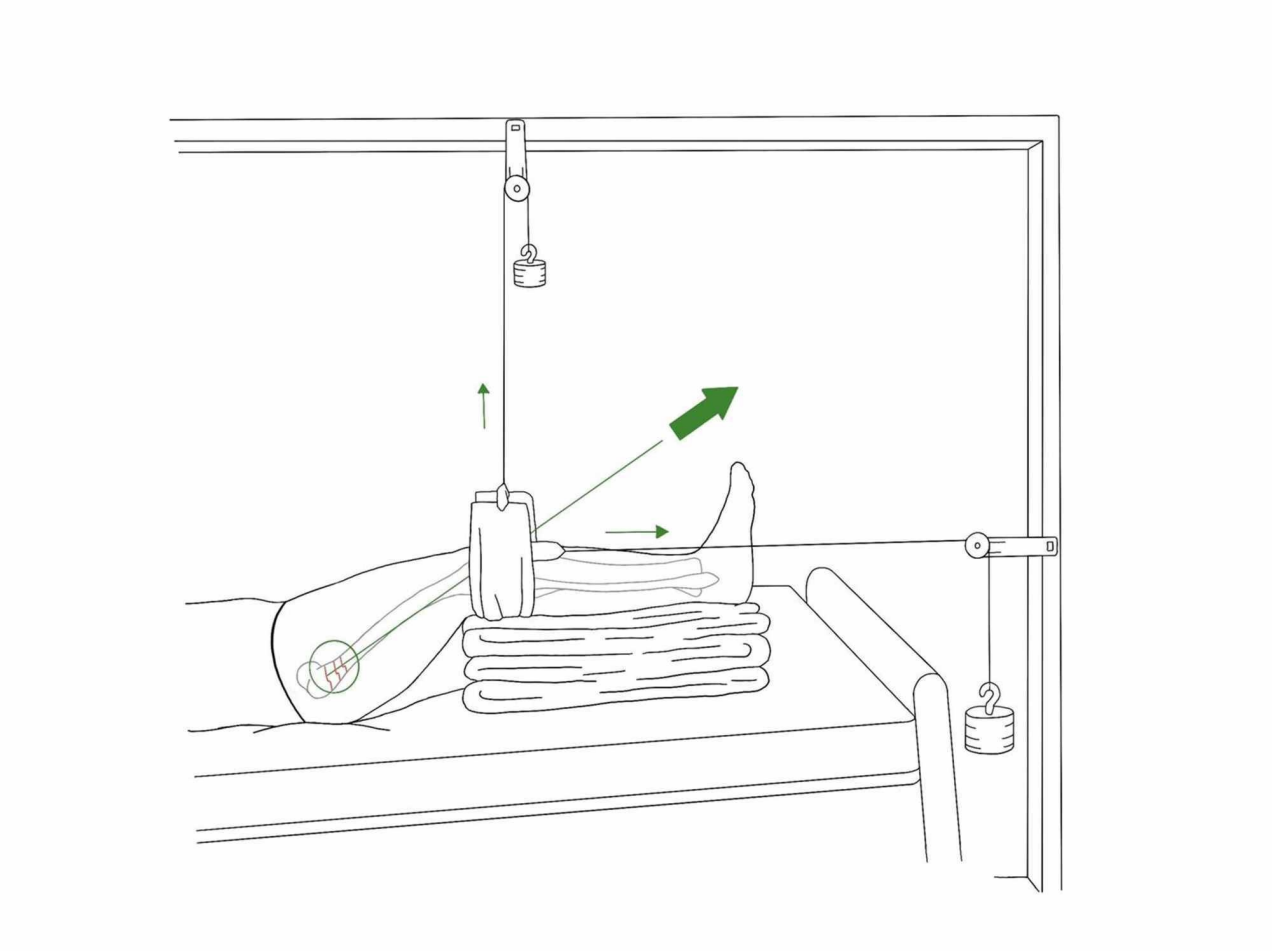
Hamilton-Russell skeletal traction via tibial pin Illustration courtesy B. Leung

If a skeletal traction is used instead, traction is through a proximal tibial skeletal pin (application described later). The bandage is first applied to the ‘U loop’ and secured. The lower leg is then carefully placed on the prepared U loop. The U loop and stirrup are passed over the pin, and traction cord is tethered to the stirrup, giving a vertical pull via a pulley system (Figure 5) [3].

Bryant’s Traction

**Figure 6:**
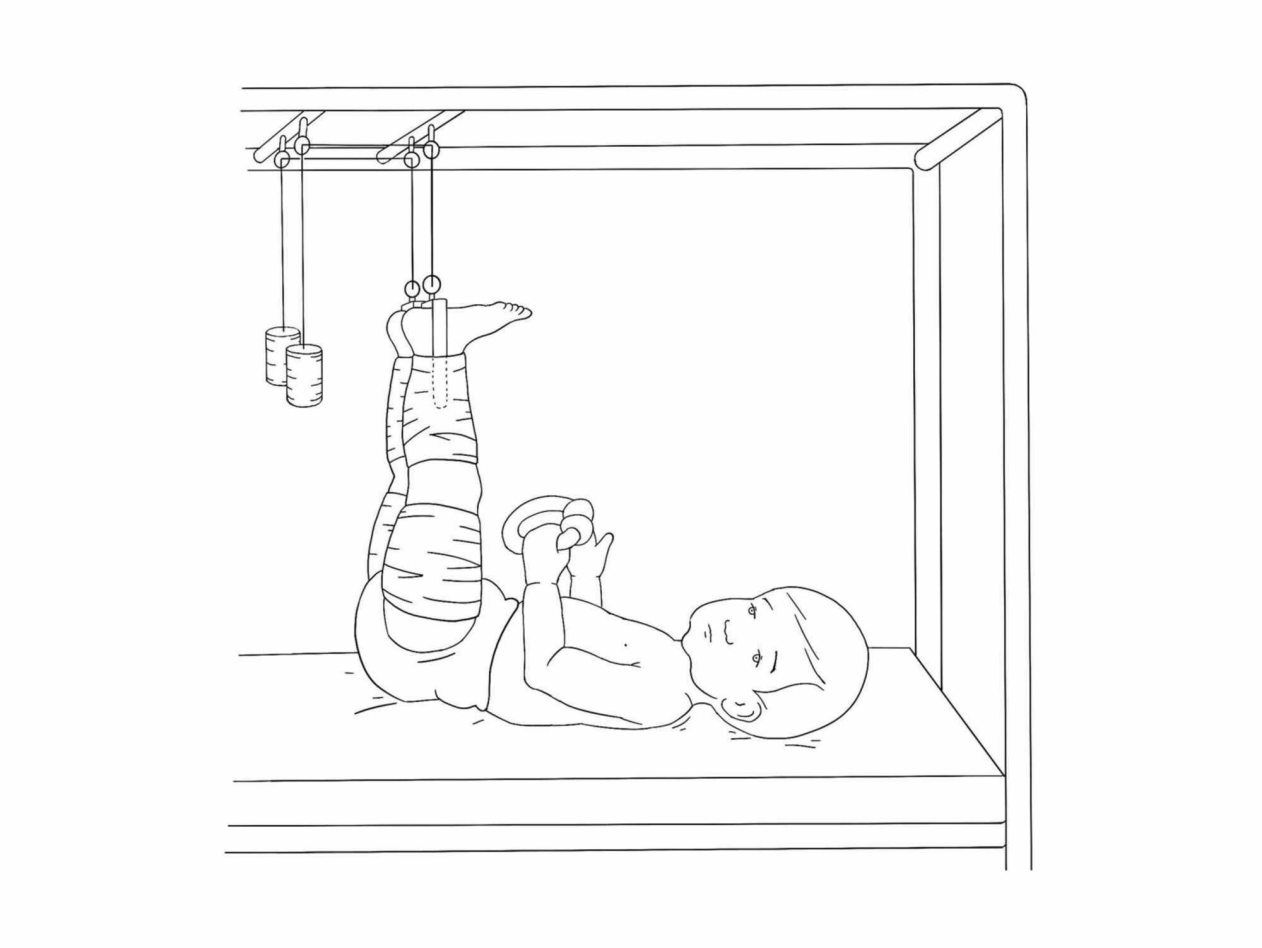
Bryant's skin traction Illustration courtesy B. Leung

A fixed traction treatment for femoral fractures in children up to the age of 18 months or less than 16 kilos. Traction is exerted through full-length extensions to both legs. The desired position is when the hips are flexed to 90 degrees and both legs suspended vertically with knees in slight flexion. The child’s buttocks should be raised so that it is just off the mattress, allowing a flat hand to pass underneath them (Figure 6). Non-adhesive skin tape is set to both legs, foam padding is placed over the malleoli, leaving a gap between child’s foot and end of extension set. Bandages are applied in a spiral fashion to prevent the extension set slipping. The cords are attached to the two beams at the top of the cot and secured; if weights are applied then pulleys need to be secured to the top beams. Weights prescribed are approximately 450 g per year of child’s age.

It is important for practitioner/nursing staff to be wary of weights tethered to the cord, which should be out of the child's reach. Additionally, small meals should be given initially to prevent distension and vomiting as the child adjusts to the position. The skin over the malleoli, dorsum of foot, and behind the knee should be regularly checked to monitor for break down and calf ischaemia can ensue hence why in the initial period, traction and bandages are released once or twice a day to allow for blood supply to legs [3,8,9].

Overhead traction is maintained for three weeks after which traction is removed, and hip plaster spica is applied with knees in 10-15 degrees flexion and foot in neutral position. The child is allowed to walk after six weeks in cast [9]. 

Cervical

Cervical injuries are either treated with open reduction and internal fixation or conservatively managed with a hard collar. Traction may be considered in patients who are not suitable for general anaesthesia, as a temporary measure or in facilities with low resources. The main use of skeletal traction is to correct and maintain the position of fracture-dislocation of the cervical spine or act as a splint for undisplaced cervical fractures.

Halter's Traction

**Figure 7:**
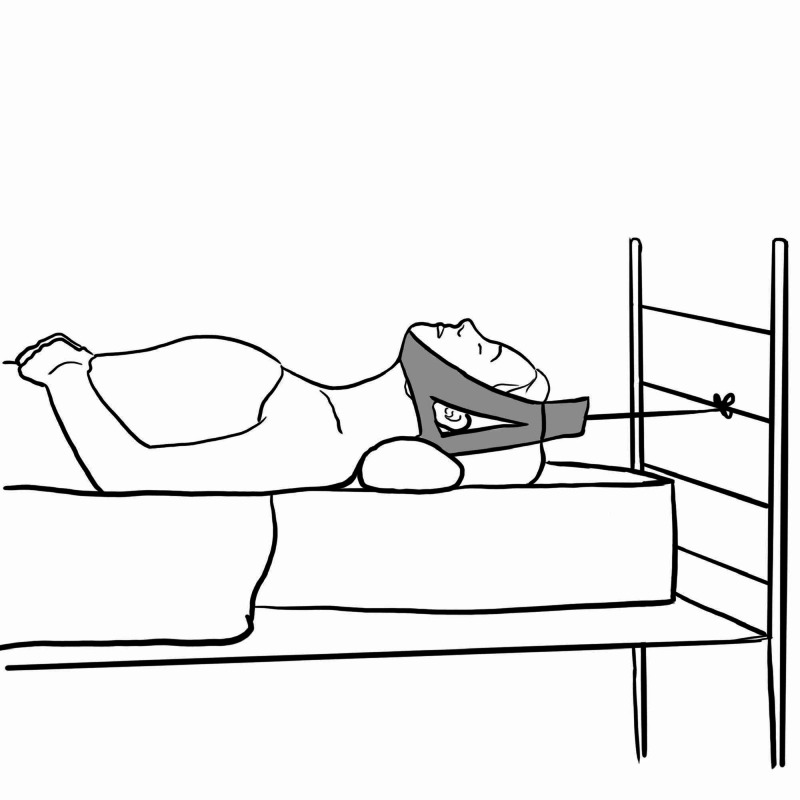
Halter's traction Illustration courtesy E. Filips

Used as a balanced traction treatment of cervical spondylosis, or torticollis. A chin type stirrup is attached to a cord which is tethered to a maximum weight of 1.4 to 2.3 kgs. The head end of the bed should be raised to provide counter traction (Figure 7). This is used to provide temporary relief for pain for these conditions but only a limited amount of force can be applied with these devices [10,11]. If used for a longer period, it can cause serious skin necrosis below the jaw [11,12].

Gardner-Wells Tongs

Application of skull callipers is generally regarded as a preliminary procedure in the context of the cervical injury [12]. The callipers are utilised in cervical dislocations/injuries and the counter-weight is dependent on the level of the injury. 

Several skeletal instrumentations have been developed over time: tongs of Crutchfield (1933), Cone (1937), Barton (1938), Vinke (1948) and Merle d’ Aubigne (1958). These tongs were applied to the skull above and in front of the ears which had the advantage of not penetrating deeply into parietal bone [12]. However, some designs require predrilling and had significant complications include haemorrhage, loosening, cellulitis of scalp, osteomyelitis of skull, cerebral abscess, calliper slippage, trismus or asymmetrical positioning [12].

Gardener-Wells tongs appeared to significantly decrease the risk of cranial and brain tissue complications due to improvement of shape which didn’t require placement close to vertex and has a tapered-pin design to control pressure to allow greater force without penetrating the inner table of the skull [13].

**Figure 8:**
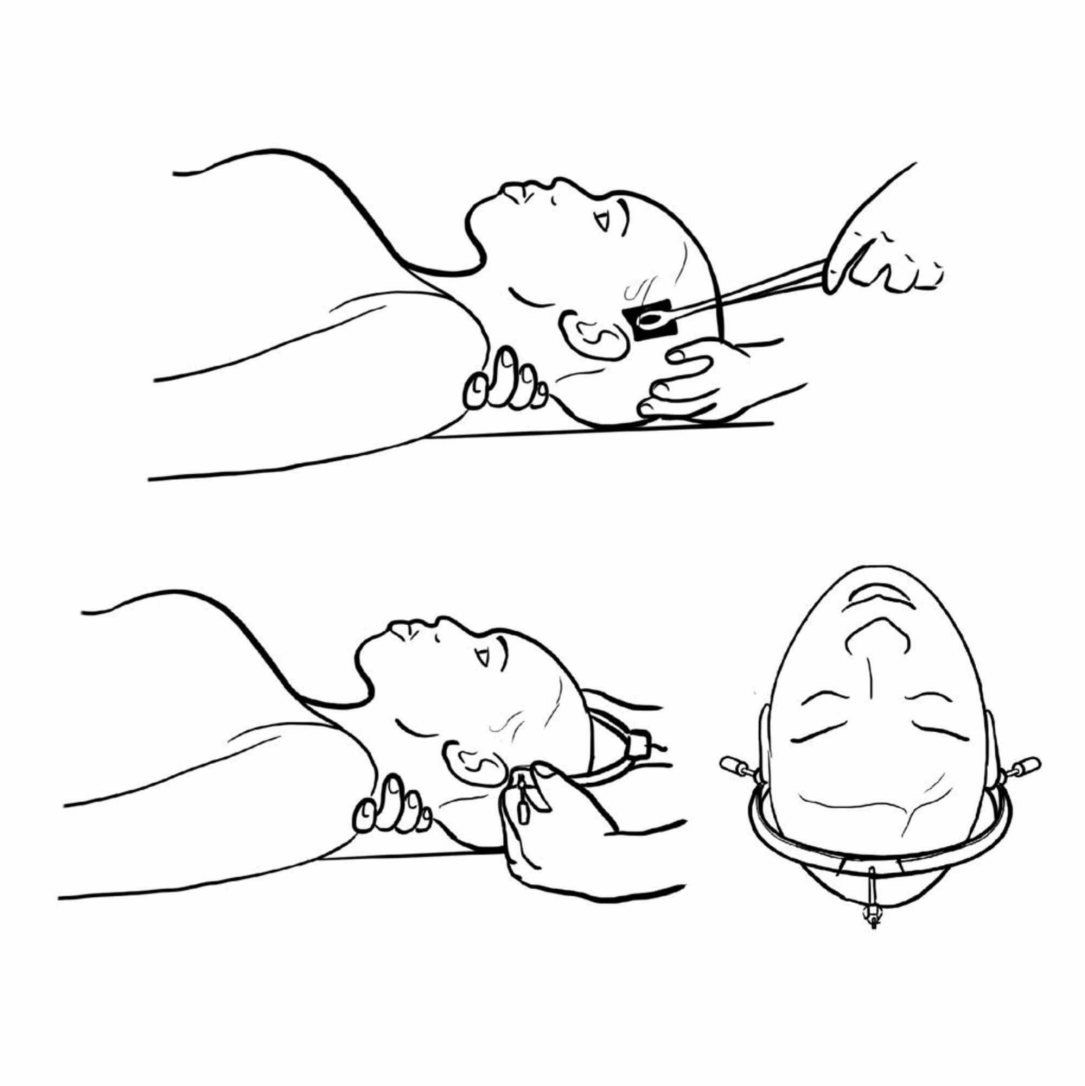
Application of Gardner-Wells tongs Illustration courtesy E. Filips

**Figure 9:**
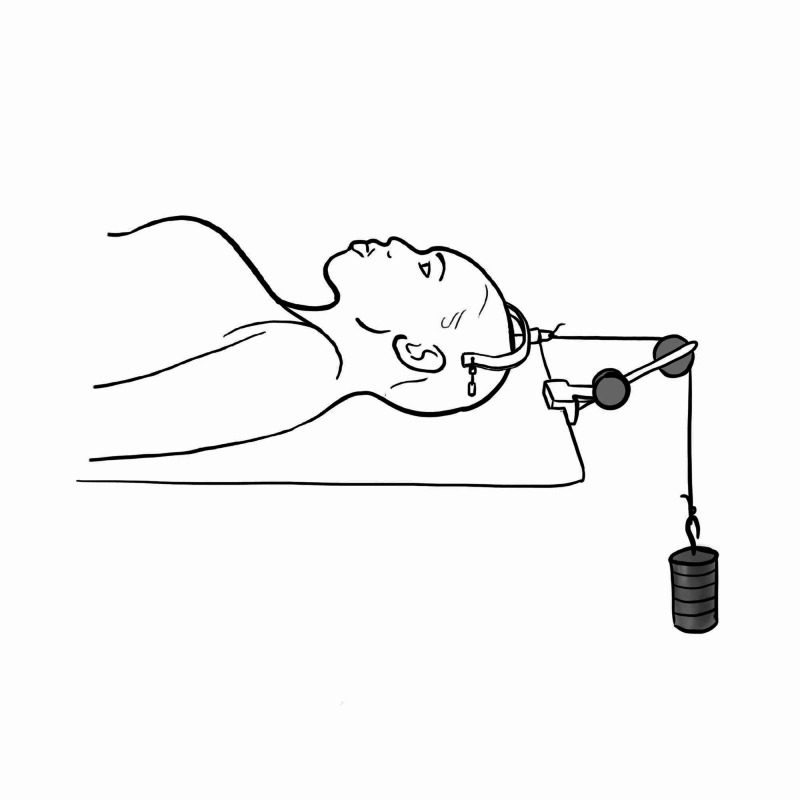
Gardner-Wells tongs Illustration courtesy E. Filips

Preparation involves shaving the scalp locally, infiltrating skin and periosteum with local anaesthetic with the patient sedated. Gardner-Wells calliper doesn’t require a scalp incision so once anaesthetised the sharpest point of the screw is advanced through the scalp to grip the outer cortex of the skull (Figure 8). These are placed at 1 cm above and in line with the pinna bilaterally, pins placed anteriorly to pinna will place the head in relative extension and alternatively in placing pins posteriorly to pinna will place the head in flexion. Recumbent or reverse Trendelenburg position to enhance tensile forces with body mass acting as counter-traction (Figure 9). Weight is adjusted according to type and level of injury. Tongs are applied for cervical facet dislocation, 4.5 kg load is initiated followed by a sequential increase of 4.5 - 6.8 kg every 5-10 minutes, and monitored carefully with serial lateral cervical spine radiographs for neurological compromise, spinal alignment and occipitocervical disassociation. Higher loads are required for lower cervical spine and unilateral facet dislocations where a total weight of as much as 63.5 kg can be used. Hangman’s fractures can require up to 2.3 to 6.8 kg to stabilise the injury, followed by halo-vest immobilisation [13].

Halo Traction

A skeletal traction device to immobilise upper cervical injuries by means of a halo, halo traction is particularly used in cervical facet dislocations, traumatic spondylolisthesis of axis (Hangman’s fracture), and combination of C1 and C2 fractures.

This device is a ring that surrounds the head with 1-2 cm air gap attached via pins to the outer portion of the skull. The halo vest or halo-gravity traction allows for healing of damaged spinal region, and also allows patient to, lie, sit, or stand. Together, this apparatus provides stability to cervical column whilst allowing patient mobility. Halo traction can be considered for treating these fractures definitively [13-15].

**Figure 10:**
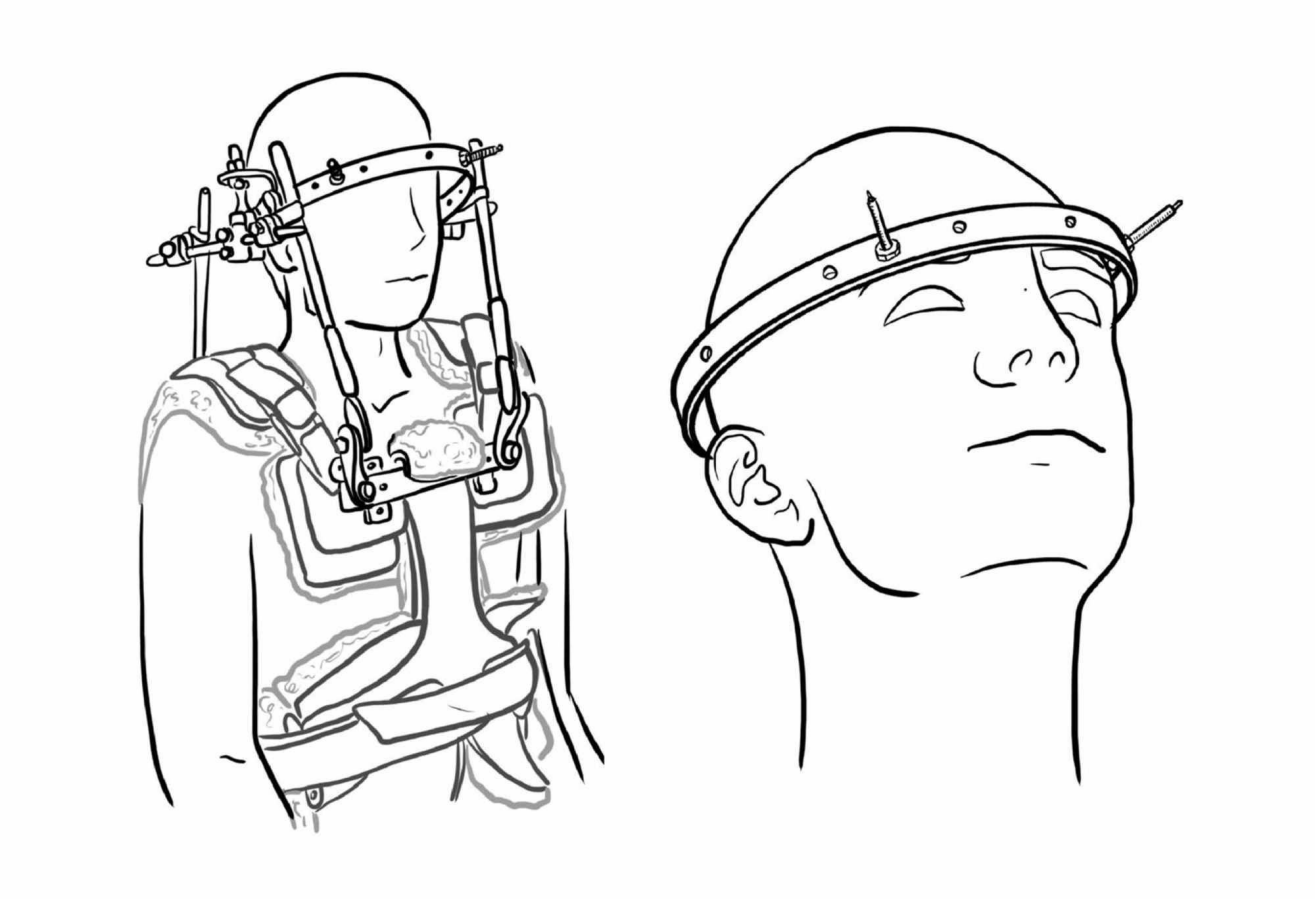
(a) Halo vest (b) halo ring Illustration courtesy E. Filips

In adults, it is a four-pin construct (Figure 10); two anterior and two posterior with an 8 inch-pounds of torque, whereas in paediatrics six-to-eight pin construct is used with lower 2-4 inch-pounds torque providing adequate stability [13,15].

Computer tomography (CT) scans may be utilised specifically to help plan pin placement in children to facilitate avoidance of cranial sutures and thin skull regions and to limit complications. Imaging modality is also relevant in trauma to rule out skull fractures before placement of pins. Other contraindications include children under two years due to risk of dural penetration and occipitocervical dissociation hence why application of light traction 4.5 kg and a lateral radiograph is performed in the setting of trauma [13]. Severe cachexia, severe scoliosis, ankylosing spondylitis, morbidly obese, elderly, non-compliant or tetraplegic patients are other factors not indicated for halo [14].

The procedure can be performed under local anaesthesia infiltration at the safe zones (Figure 10). The anterior pin is sited 1 cm just above the lateral 1/3 of the orbit (eyebrow). Skin incisions are made at the proposed sites at the screw hole of halo and anterior pins are placed through with eyes tightly shut. If not, the patient will not be able to close eyes due to ‘tenodesis’ effect of the pins on the orbicularis muscle. Anterior sites are lateral to supraorbital and supratrochlear nerve hence if sited too medially can cause nerve damage, or risk dural leak with subsequent meningitis or brain abscess if too deep in the frontal sinus. The posterior pins are placed 180 degrees from anterior pins, above the level of the pinna. The pins are initially tightened with finger and then tightened with torque-limiter screwdrivers. Locking nuts are then placed over each halo [15,16].

The system is made from carbon fibres to make it compatible with magnetic resonance imaging (MRI). However, it severely limits the visual field by restricting head movement and therefore patients need to be advised and accustomed to turn around to see behind or beside them.

This device is usually removed after three months, following the radiological confirmation of the objective. Subsequent removal of halo, a neck collar is provided to support for neck muscles that have become deconditioned and weak, making the head feel heavy [14,15].

Complications of up to 68% following halo application have been reported, include pin loosening, dislodgment and infection. Pin tightening should occur at 24 hours and one week and pin infections should be managed with local pin care and occasional oral antibiotics; however, for severe infection pin replacement is required^ ^[14,15].

Forearm/distal radius

Finger Trap Traction

**Figure 11:**
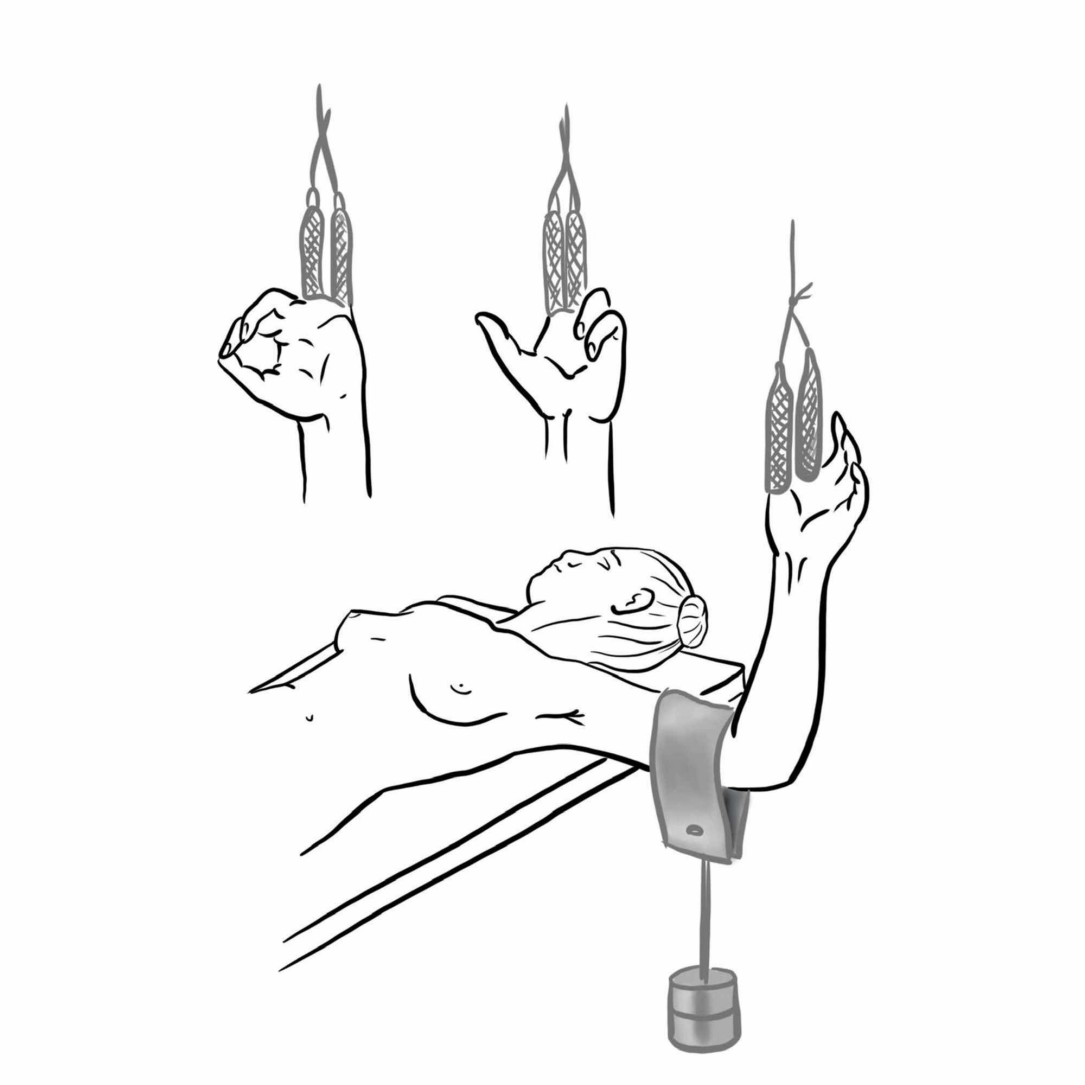
Finger trap traction Illustration courtesy E. Filips

First described by Caldwell in 1931, this method involves inserting the digits into finger traps, ensuring they are well secured, and then suspending them onto a drip stand or equivalent with the elbow flexed to 90 degrees; the additional weight is hung over the humerus to provide the traction to disimpact the injury (Figure 11). Normal anatomy is re-established via ligamentotaxis and reduction under gravity. This technique sometimes can be used to assist in the application of splints/casts or a combination of both [17].

Elbow

Dunlop Traction

John Dunlop of Pasadena, California originally described this balanced traction system for midshaft or supracondylar humerus fractures. This is indicated in those with gross instability/ inability to achieve reduction through manipulation, in whom there is no palpable radial pulse at the time of presentation, or in whom when the fracture is reduced, the pulse disappears and extension of the elbow is sufficient to allow a return of the pulse but results in the fracture slipping again [18].

The original description describes traction under heavy sedation with morphine, aimed at gradually straightening the arm, alternating with the traction in a gradual process of reduction. He aimed to achieve a complete reduction in 24-36 hours [18]. Any tendency for varus angulation can be controlled by placing the forearm in pronation and conversely any tendency for valgus angulation can be controlled by placing the forearm in supination. Elevation of that side of the bed is an essential part of the management [19].

**Figure 12:**
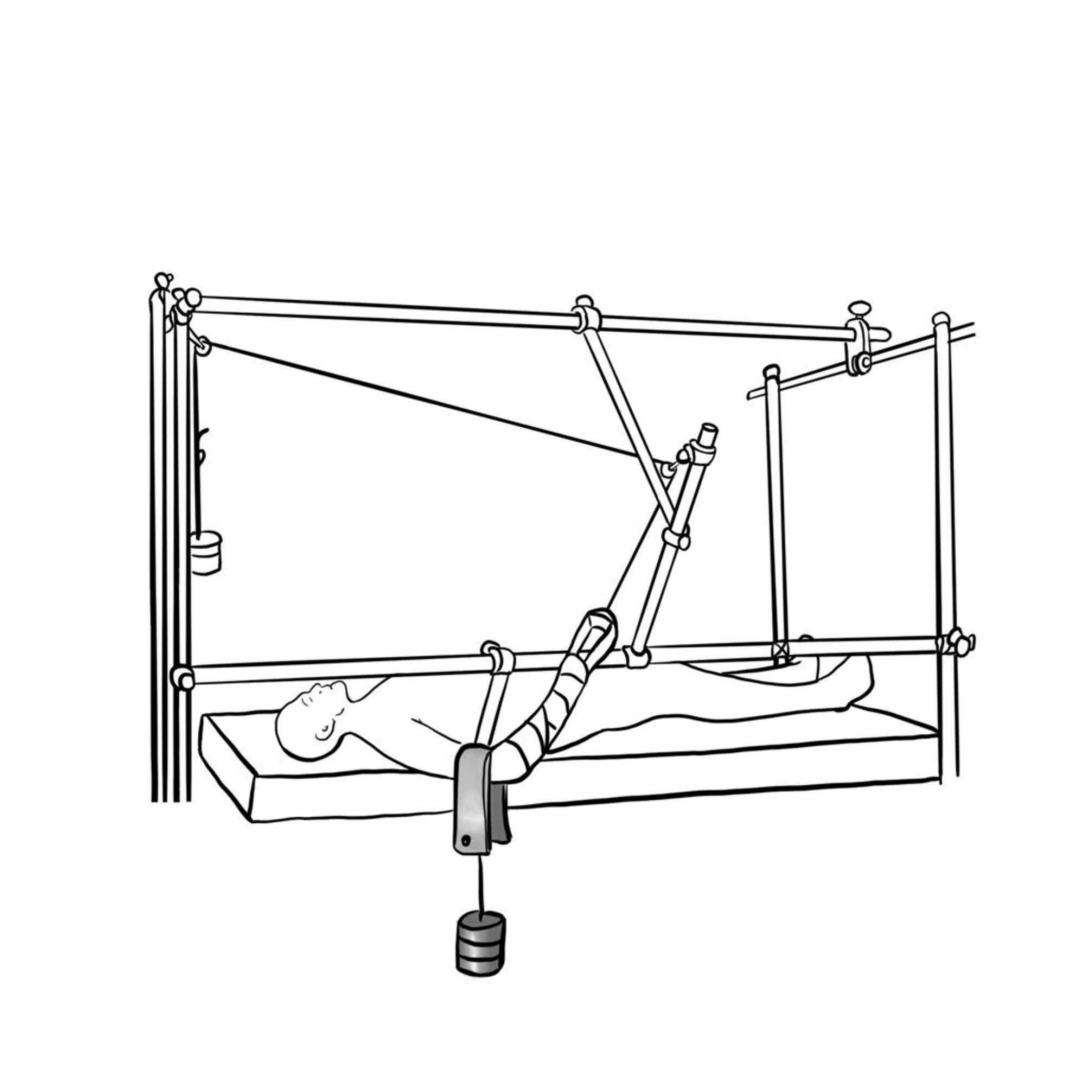
Dunlop traction Illustration courtesy E. Filips

The vertical counter-traction for the humerus, proximal to the fracture site, has usually been achieved by a wide piece of non-adhesive felt to which a 1.5-kilogram weight is applied and forearm traction with 1-kilogram weight on upper arm elbow flexed at 45-60 degrees (Figure 12). One should check the X-rays taken and if necessary further gentle manipulation is carried out with further X-rays. The procedure is usually done under general anaesthesia [19].

When sufficient callus is visible at three weeks, traction is removed and the arm is gradually brought to a right angle, and a plaster splint or sling is applied.

Christopher Colton & Fergal Monsell emphasised that these are overtreated with surgery and advocate considering less invasive procedures such as traction for displaced supracondylar humerus fractures, particularly where image intensification is not available [20].

Skeletal Tractions

Skeletal traction is indicated for those with shortened unstable fractures/dislocations of the extremity. This is particularly relevant for the lower limb where it may be difficult to immobilize with splinting alone or require greater force than what skin traction could provide [21,22].

**Figure 13:**
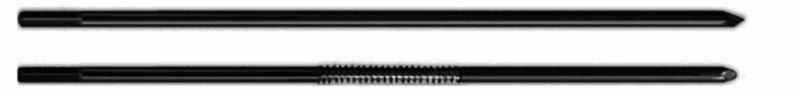
Traction pins

Kirschner wires (K-wires) and Steinmann pins are used as traction pins. Those that are threaded (eg. Denham pins) are less likely to loosen than smooth implants but tend to bend. The diameter of the pins is determined to be 1/3 of the width of the bone it is placed in (Figure 13). Required maintenance weights are roughly estimated at 1/10-1/7th of the patient’s body weight [21].

Femur

Distal Femur Traction (Skeletal 90-90 Traction)

**Figure 14:**
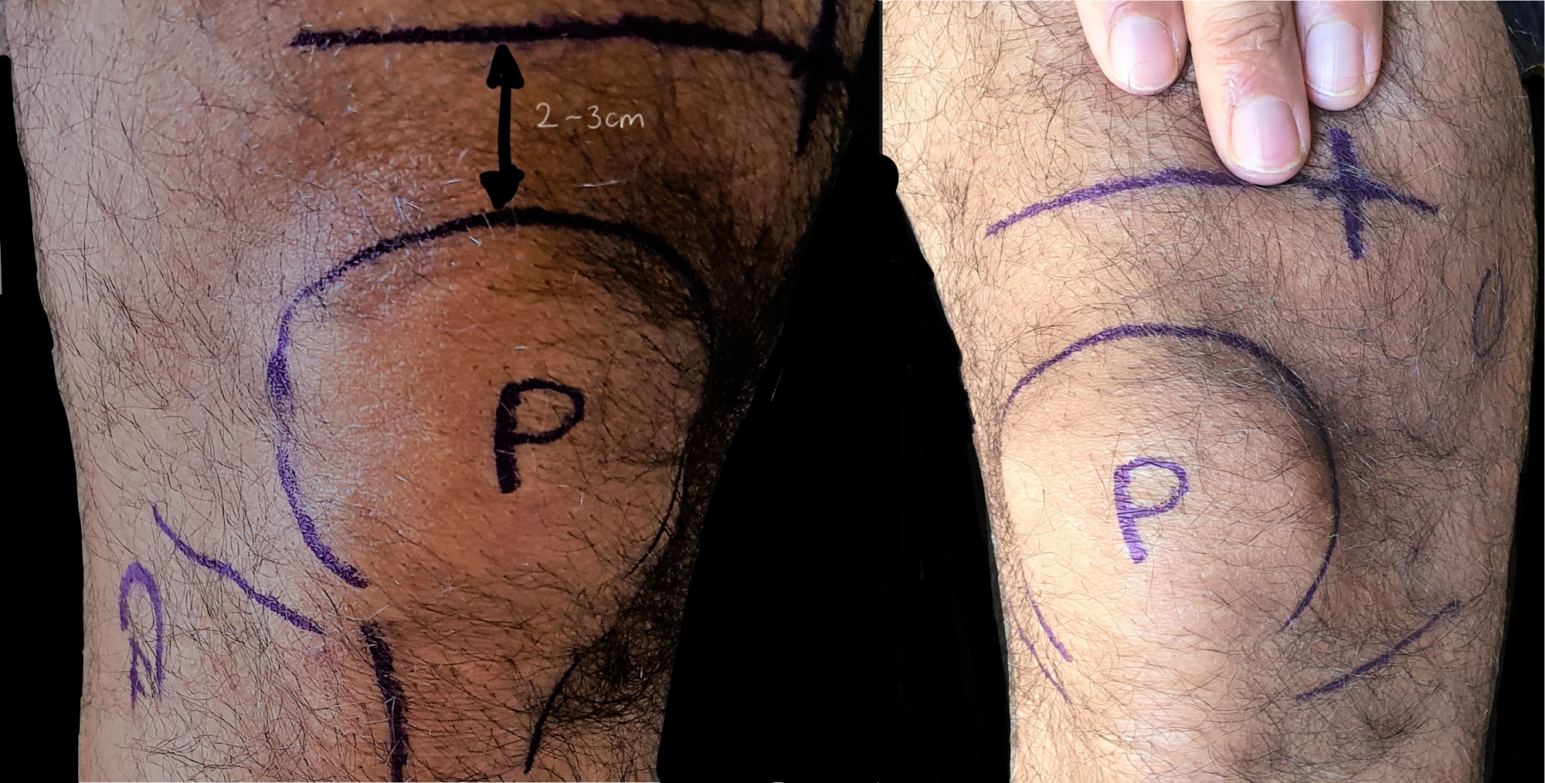
Femoral pin traction P: patella; F: fibula head; O: adductor tubercle; +: Pin insertion site Photo courtesy K. Dhaliwal

This is indicated for unstable hip dislocations, acetabular, proximal femur and shaft fractures. Traction pin placement is placed at the metaphyseal-diaphyseal junction of the femur. Prior to insertion of Steinmann pin, palpate the superficial landmarks: patella, joint line, and adductors tubercle which allows for identification of the placement and limits complications. A transverse line 2 cm proximal from superior pole of patella is marked and then palpated from midline of the mark two to three finger breaths medially to mark an intersecting line in the sagittal plane, which should be slightly proximal to the adductor tubercle (circle) (Figure 14).

Once identified, local anaesthetic is infiltrated superficially and then deeper into periosteum. The skin incision is made and blunt dissection is made down to the bone, the track is developed with the aid of artery forceps or clamp. Pins are placed medial to lateral with the knee in flexion, the pin is then walked onto the medial femoral condyle to ensure central placement and confirmed on fluoroscopy; the drill is then attached to the pin and is advanced in perpendicular to the bone in axial plane and parallel to the marked line until skin tenting is noticed on the lateral side.

**Figure 15:**
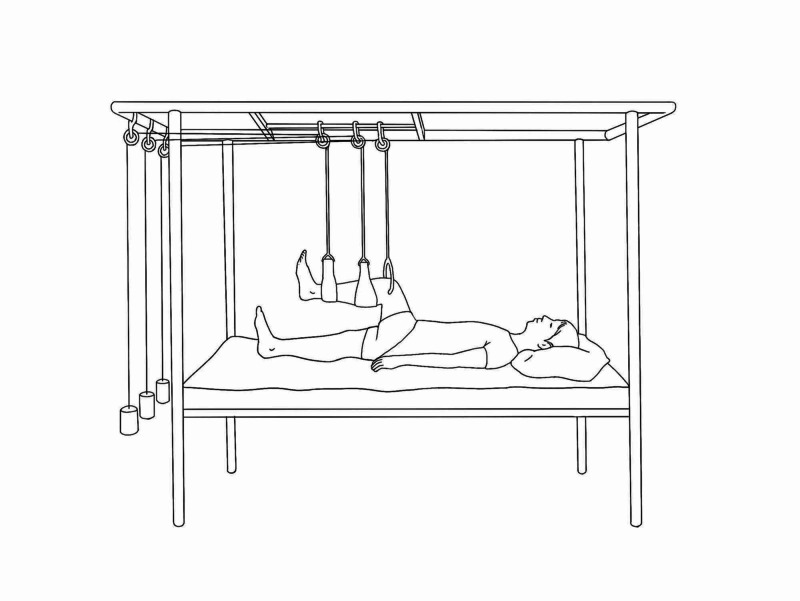
90-90 skeletal traction Illustration courtesy B. Leung

Local anaesthetic is infiltrated and a counter incision is made. Once radiologically confirmed satisfactory placement, traction bow is secured to pin and 9-14 kg of traction that is hung off the side of traction bed and the lower leg is supported with U-Loops (Figure 15) [16,22,23].

Distal femoral traction pin is considered when the knee joint is injured or its stability is unascertained.

Proximal Tibial Traction (Perkins)

Proximal tibial traction is indicated for femoral shaft or subtrochanteric fractures and is generally easier to apply in obese due to ease of palpating the landmarks. The landmarks palpated and marked include patella, patellar tendon, tibial tubercle, joint line and head of the fibula.

**Figure 16:**
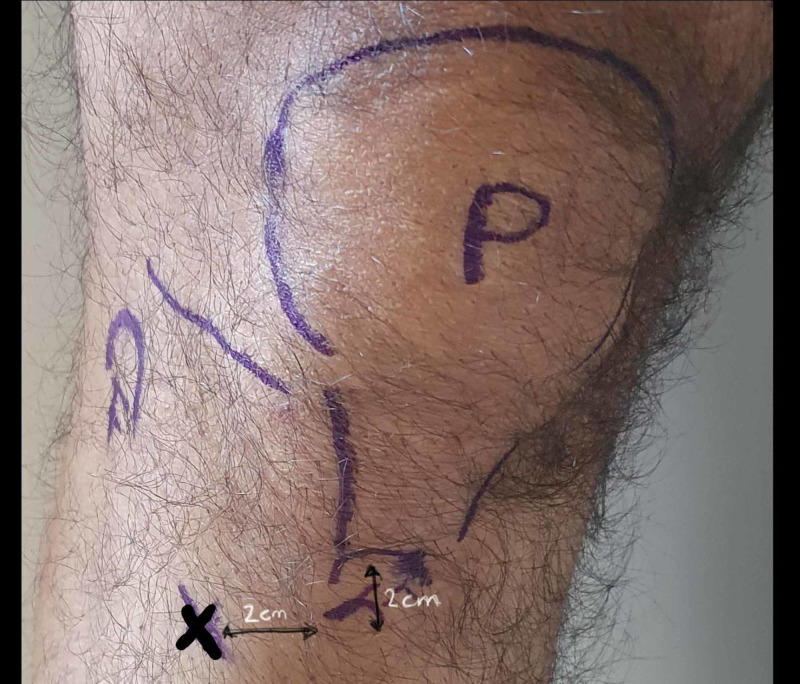
Surface marking for tibial pin P: patella; F: fibula head; O: adductor tubercle; X: pin insertion site Photo courtesy K. Dhaliwal

This site of insertion is 1-2 cm or one to two finger breaths distal and 2-3 cm or two-finger breaths lateral to tibial tubercle.

**Figure 17:**
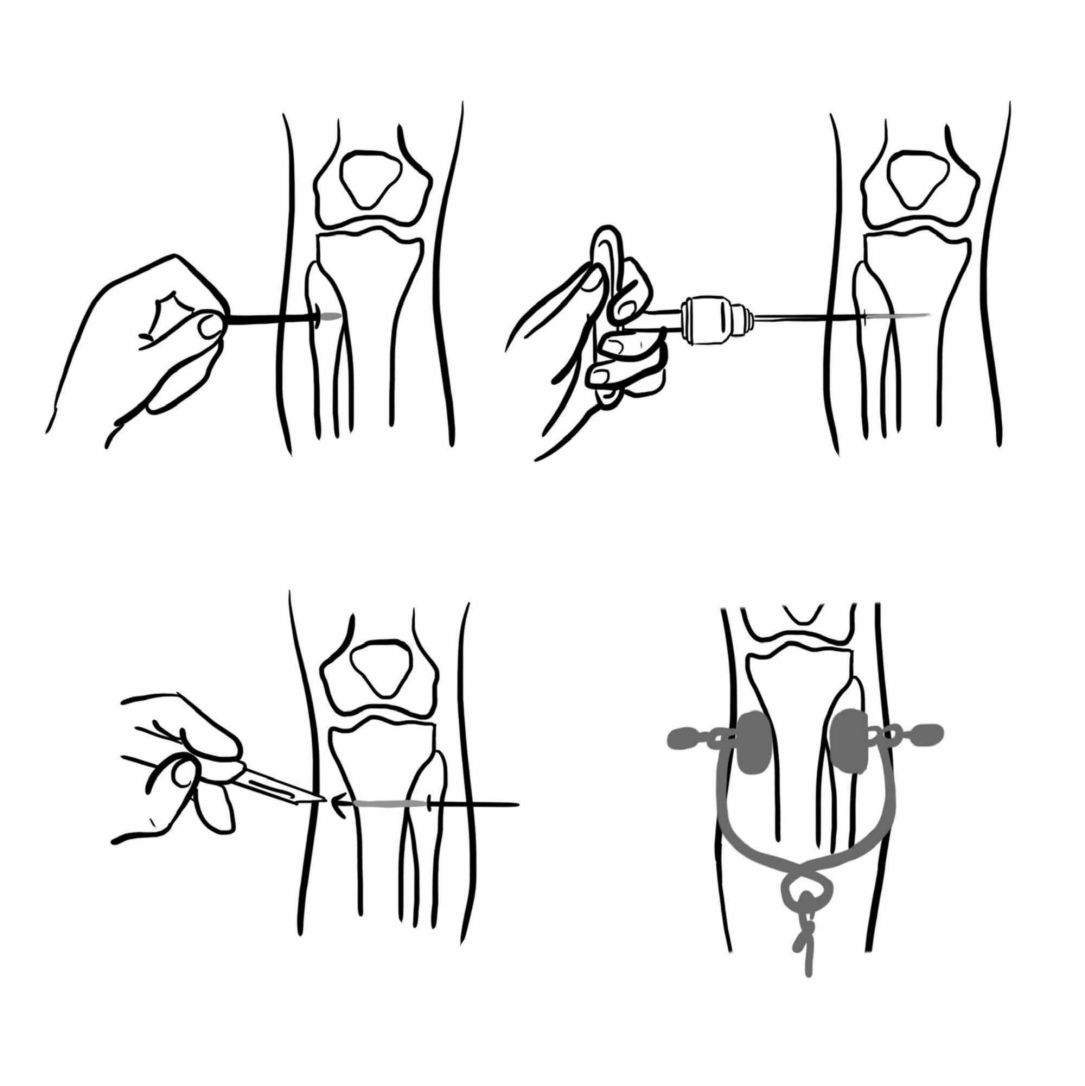
Steps in applying tibial pin Illustration courtesy E. Filips

**Figure 18:**
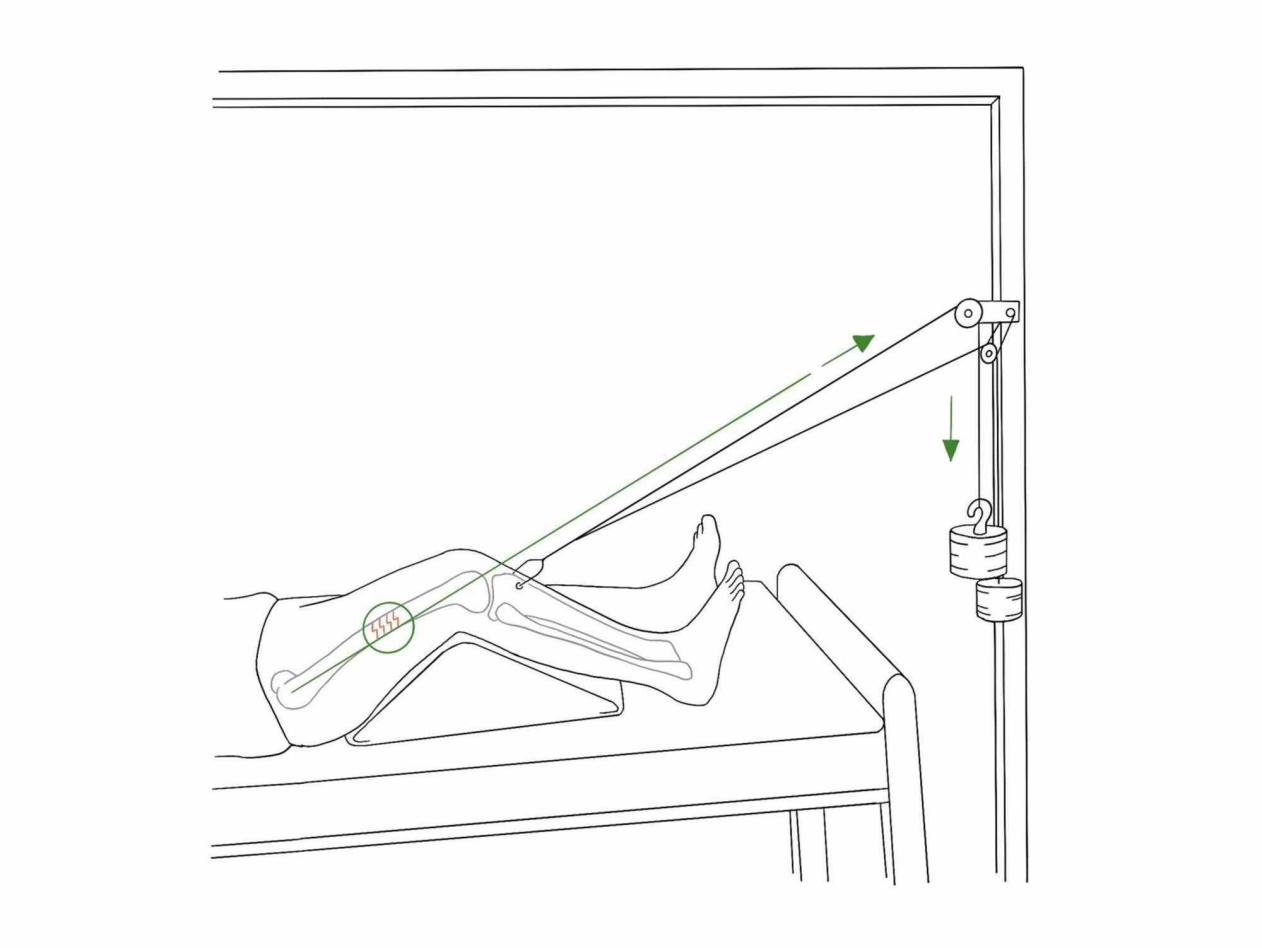
Perkin's traction Illustration courtesy B. Leung

Once identified and appropriately local anaesthesia infiltrated, a skin incision is applied in line of axis of the bone. The pin is placed from lateral to medial to avoid causing iatrogenic damage to the peroneal nerve. As the tibia has a triangular cross-section, the pin may not be initially completely perpendicular on entry. Radiographs should be taken to confirm satisfactory placement. If a Denham pin has been used, it is passed through, making a counter incision and then a stirrup is placed and secured with traction applied (Figure 17) [22,23]. The knee is supported with a triangular wedge to control the distal femur fragment which is flexed by the deforming force of gastrocnemius (Figure 18). Proximal tibial pins are not recommended in children younger than 10 years because of the potential for proximal tibial physeal injury [23]. In distal femur and proximal tibia pin traction, rotational alignment may not be corrected and hence require anti-rotation splints.

Tibia

Distal Tibial-Fibular Traction

**Figure 19:**
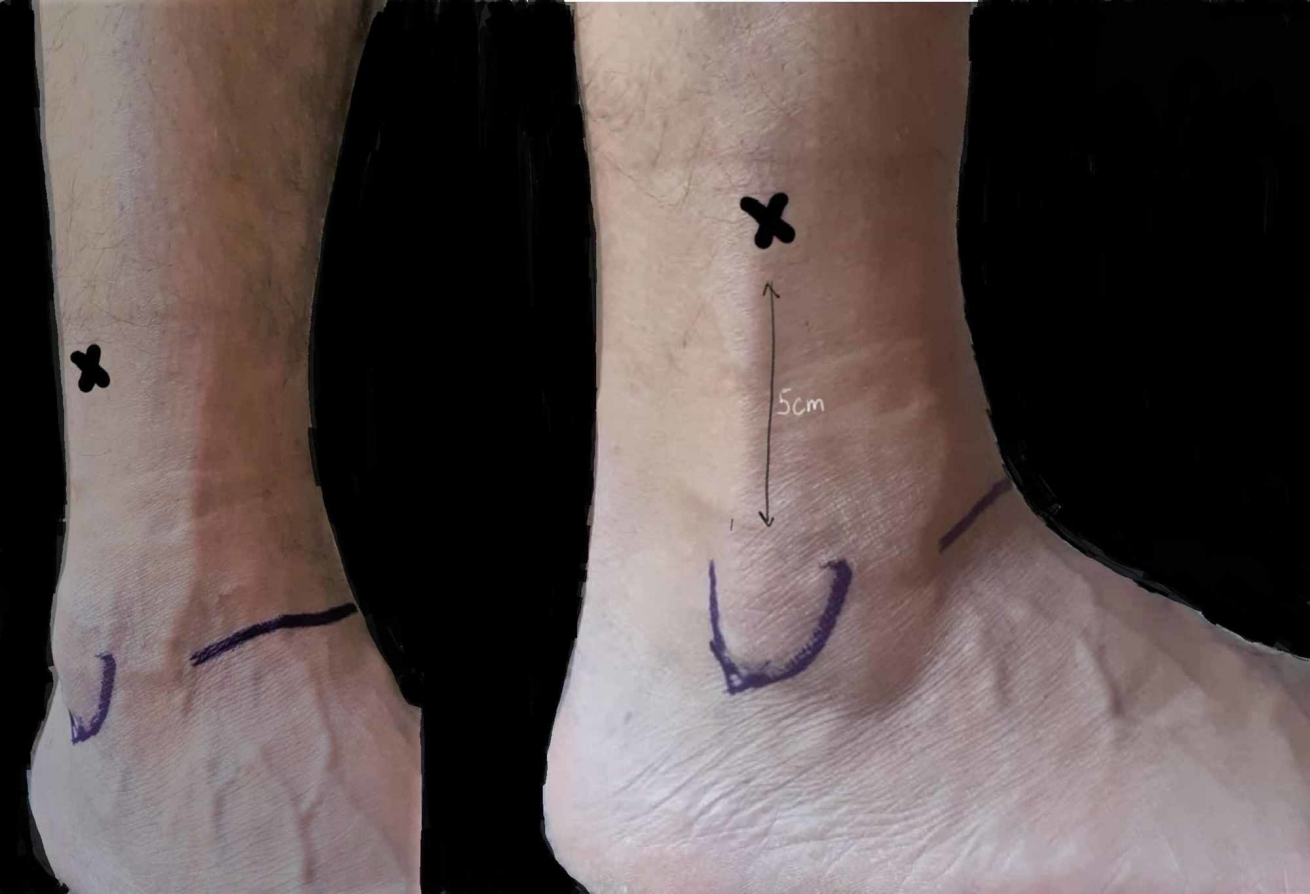
Surface marking for distal tibial pin X: Pin insertion site Photo courtesy K. Dhaliwal

**Figure 20:**
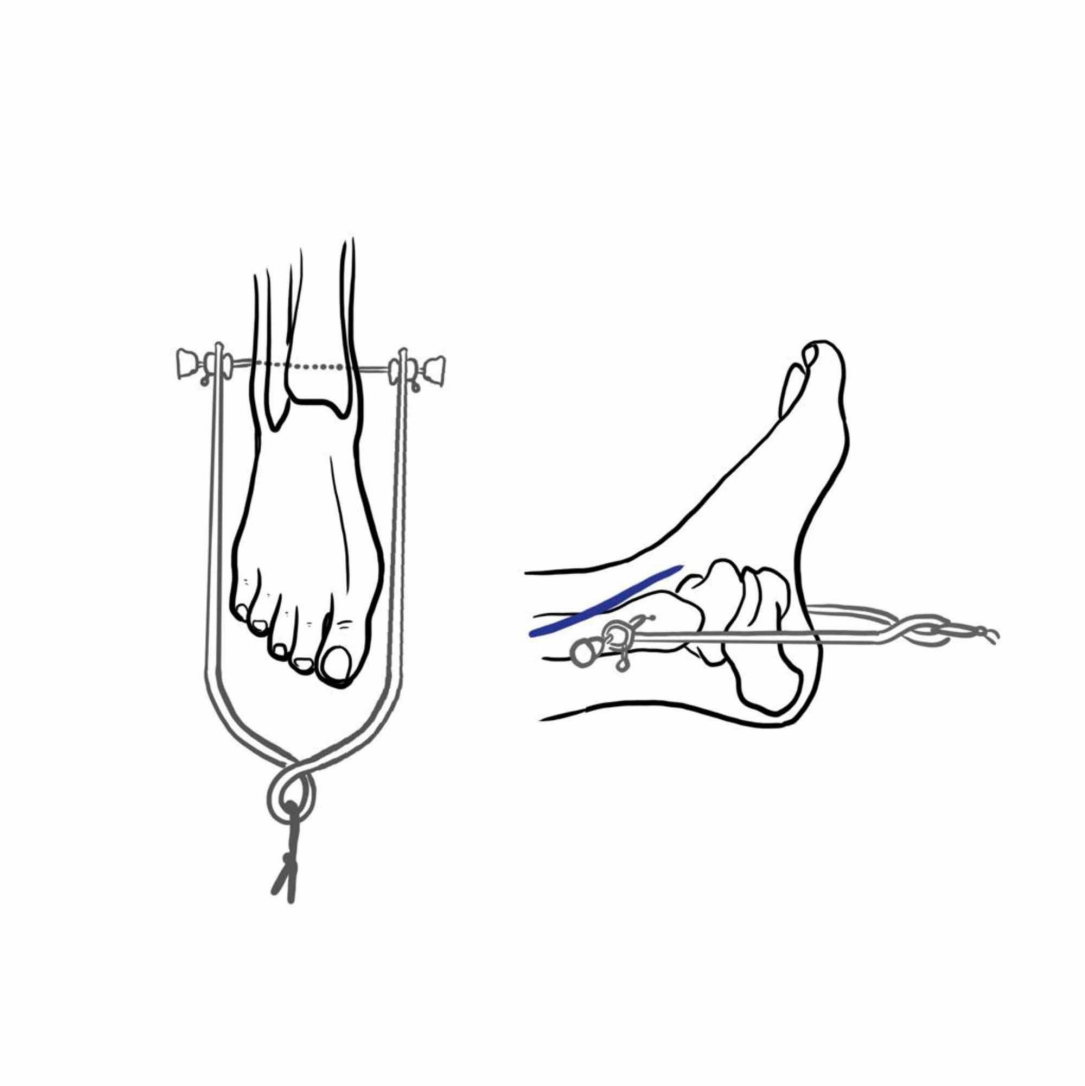
Distal tibial pin traction Illustration courtesy E. Filips

Fractures distal to the knee require more distal traction, it may be useful in setting shortened tibial plateau fractures. Pin placement is aimed to avoid the superficial peroneal nerve and intra-articular placement, therefore, a transverse line is marked 5 cm proximal to the ankle joint (Figure 19). Once the skin is infiltrated, the trajectory of the pin is similar to syndesmotic screw going from posterior to anterior via fibula, engaging four cortices (Figure 20) [22].

Calcaneal Traction

**Figure 21:**
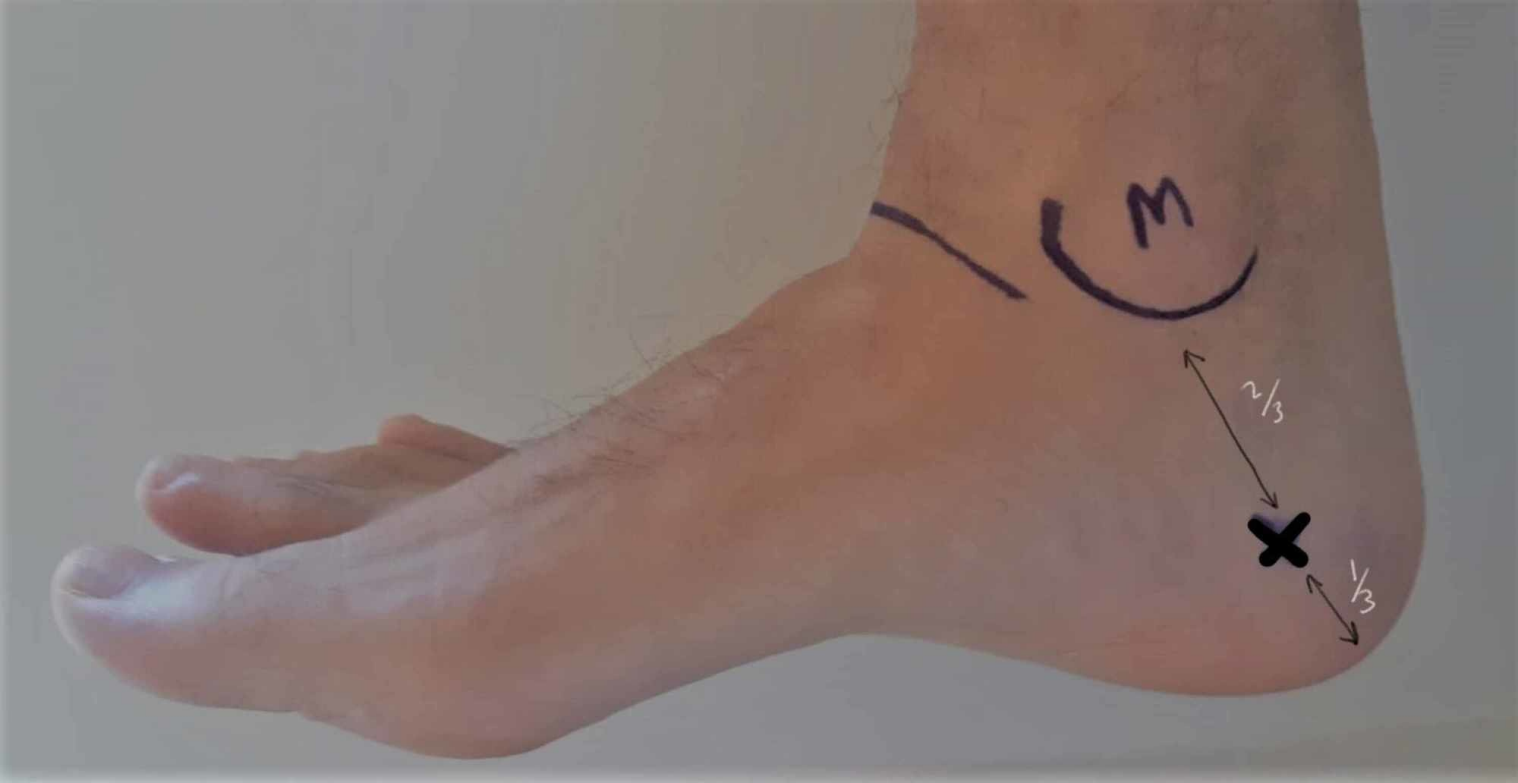
Calcaneal pin placement M: medial malleolus; X: Pin insertion site Photo courtesy K. Dhaliwal

Calcaneal pin traction is reserved for tibial shaft, pilon and subtalar fractures. Placement is from medial to lateral to avoid injury to the posterior tibial neurovascular bundle which sits posteroinferiorly to medial malleolus. Superficial landmarks which are identified and marked are medial malleolus, posterior tip of calcaneus, tibiotalar and subtalar joint. A line is drawn from tip of calcaneum to medial malleolus. The entry point is 2/3 from the line drawn from medial malleolus to tip of calcaneum (Figure 21). Local anaesthesia is infiltrated to skin and the pin is positioned in place after dissection and radiologically confirmed then the pin is advanced, finally, the counter skin is infiltrated, incision is made, pin is advanced further and traction bow is applied so weights can be applied [22,24,25]. Potential risk includes damage to medial calcaneal nerve and stiffness of subtalar joint.

Olecranon Traction

Fractures of the shaft or distal end of the humerus can be managed with skeletal traction via an olecranon pin. It is marked 3 cm distal to the tip of the olecranon, local anaesthetic is infiltrated and a skin incision is made, careful dissection down to bone using ulna nerve safety precautions is performed and small artery forceps are used to dilate track.

**Figure 22:**
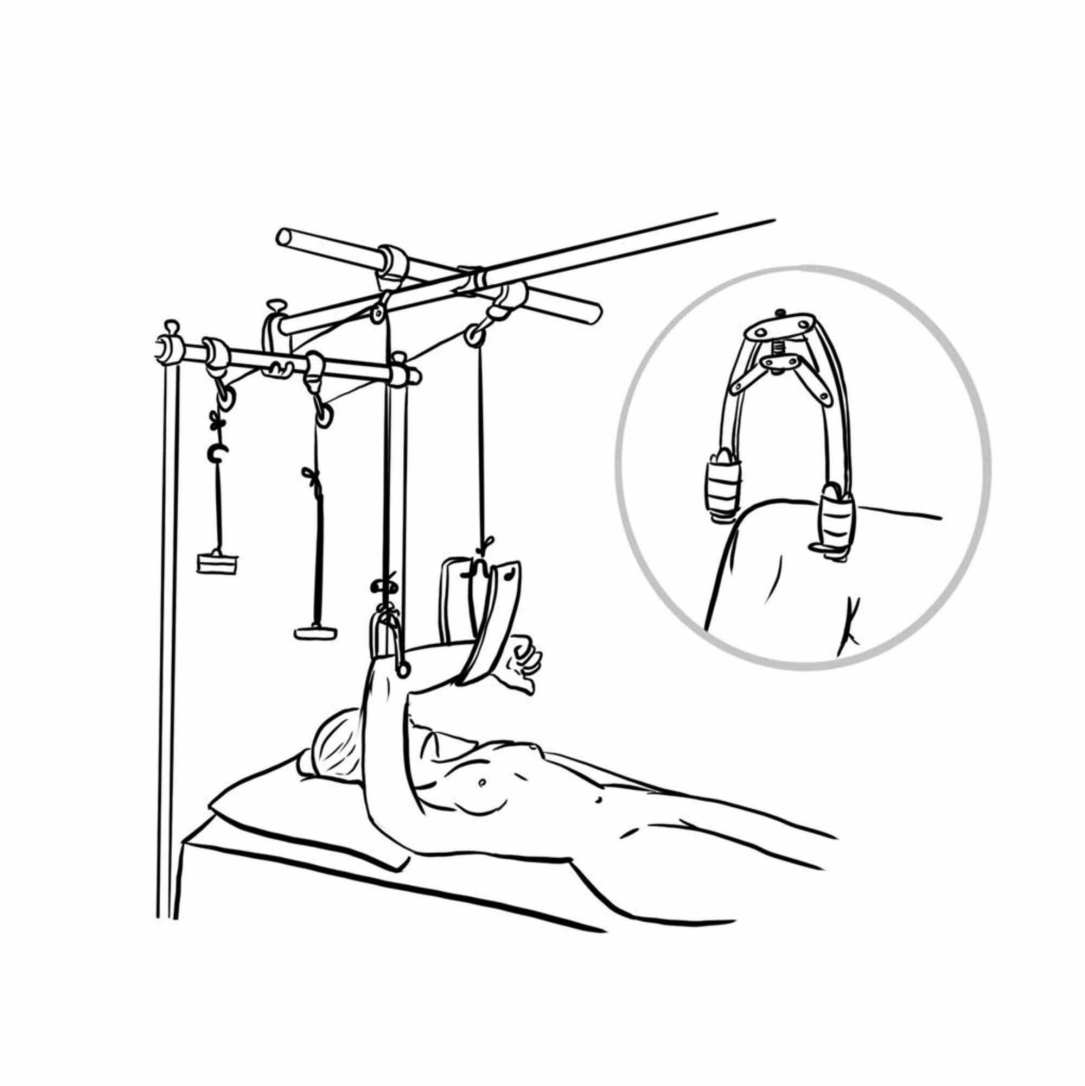
Overhead olecranon pin traction Illustration courtesy E. Filips

The K-wire is then passed medial to lateral perpendicular to the longitudinal axis of ulna confirming with fluoroscopy. The pin is assembled to the traction bow which is tethered to a cord which passes over a pulley and attached to a weight whilst the forearm is supported by slings attached to a central crossbar (Figure 22) [10,26].

Metacarpal Traction

**Figure 23:**
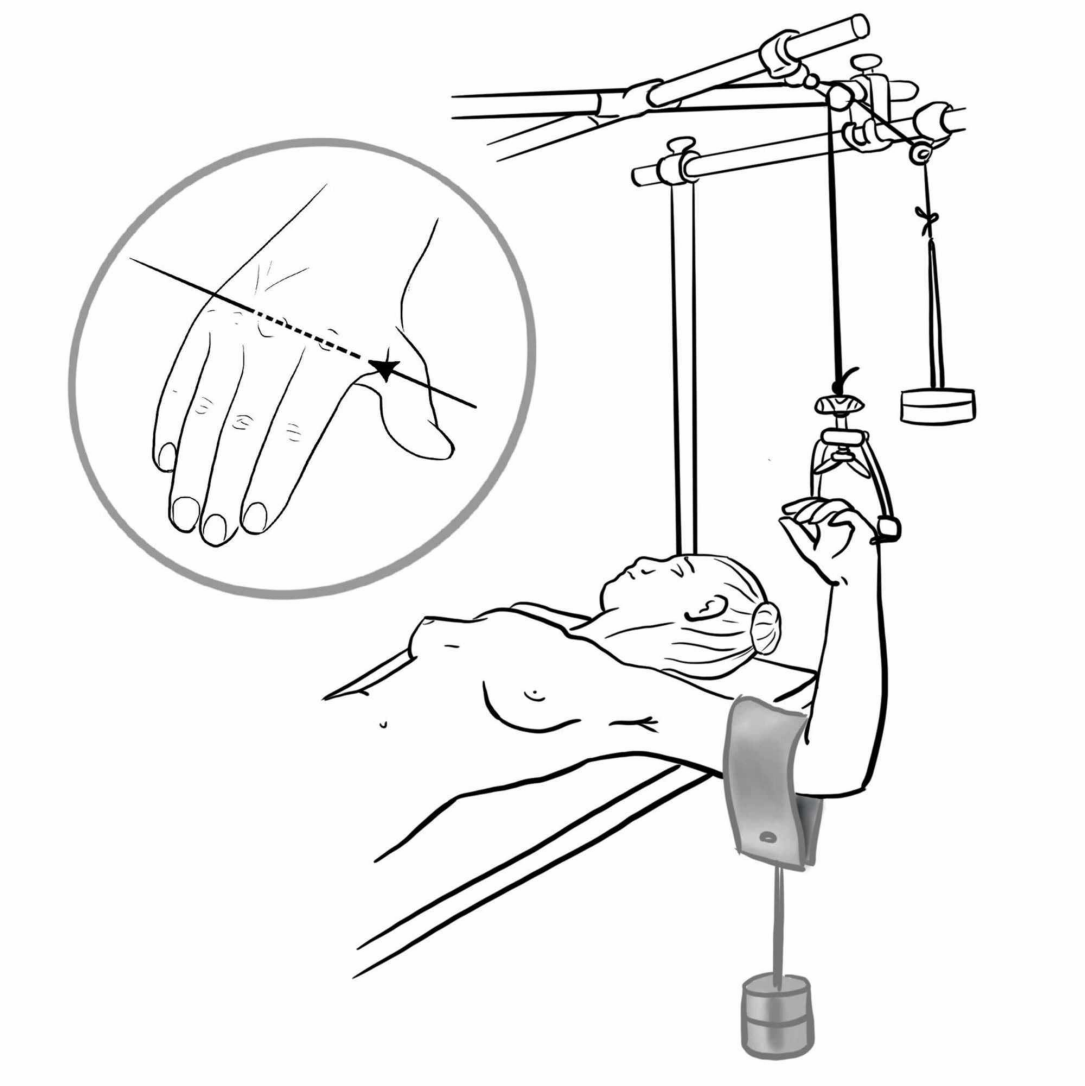
Metacarpal pin traction Illustration courtesy E. Filips

Metacarpal traction is used rarely in developed health care systems but can be applied for difficult and unstable distal radius fractures and forearm shaft fractures. K-wires are placed through metacarpal diaphysis 2.5 cm proximal to MCP joint of the index and middle finger and perpendicular to the axis of radius. A small skin incision is made into anaesthetic infiltrated skin, manually moving the 1st dorsal interossei muscle volarly. K-wire is used to palpate the bone, ensuring it is not anterior or posterior and crossing radially to ulnarly under x-ray. Structures at risk include digital vessels and nerves with stiffness to intrinsics. This can be then applied to traction bow to provide longitudinal traction (Figure 23) [10].

Skeletal pin complications and prevention

When skeletal pins are applied potential complications may occur, this includes cortical defects, which act as stress risers and may predispose to fractures, or pin site infection can develop secondary pin-tract osteomyelitis or septic arthritis if placed intra-articularly [22].

**Figure 24:**
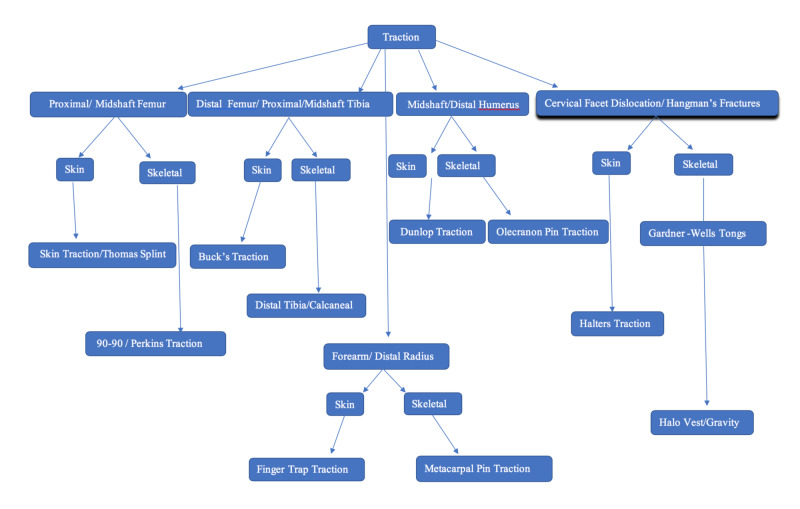
Decision-making flow chart for type of traction to apply

Hence it is of essential importance to carefully place pins while not causing iatrogenic injury. Pins should be applied transmedullary with the aid of fluoroscopy to prevent transcortical placement reducing risk of fracture. Pins advanced into the bone using drills should be pulsed with saline to reduce thermonecrotic damage. therefore minimising infection and loosening. The pin should not be driven forward and then retracted to prevent early loosening. There is a higher chance of developing pin site infection in some tractions such as femoral traction due to bulky muscle, hence chlorhexidine swabbed gauze or sponge should be placed around the pin site and to have regular monitoring of pin sites to prevent infection. Furthermore, certain tractions require specific rehabilitation to ensure stiffness doesn't develop and to avoid flexion contractures (Table 1) (Figure 24) [16,21].

**Table 1:** Indication, landmarks and danger structures of various skeletal traction

Skeletal Traction	Landmarks	Danger Structures	Indications
Distal femur	Proximal to adductor tubercle	Femoral artery (adductor canal)	proximal/midshaft femur fracture
Proximal tibia	Tibial tuberosity	Common peroneal nerve	Distal femur fracture
Distal Tibia	5cm above ankle	Saphenous vein/ superficial peroneal nerve	Tibial plateau / midshaft tibia fracture
Calcane	2/3 from tip of medial malleolus to tip of calcaneus	Medial calcaneal nerve/ sural nerve	Distal tibia fracture
Olecranon	3 cm proximal to the tip of olecranon	Ulnar nerve	Midshaft / supracondylar humerus fracture
Metacarpal	2.5 cm proximal to MCP	Digital nerves / vessels	Forearm / distal radius fracture

## Conclusions

Traction is used for immobilisation of limbs and despite recent advances in internal fixation, it remains an important technique for pain relief, keeping long bone fractures reduced and preventing joint contractures. The skills, however, are often forgotten unless one rehearses them with the same frequency and discipline as one would their routine surgical skills. We hope this provides an aid for the practitioner when the need arises, to guide them in application, planning and decision-making processes when traction is considered.
